# Thirty-Day Morbidity and Mortality After Total Knee Replacement in a Tertiary Care Hospital in Pakistan

**DOI:** 10.7759/cureus.35409

**Published:** 2023-02-24

**Authors:** Ziad Ali, Masood Umer, Shahryar Noordin

**Affiliations:** 1 Department of Orthopaedic Surgery, The Aga Khan University Hospital, Karachi, PAK

**Keywords:** knee surgery, knee replacement surgery, tka, total knee arthroplasty, tkr, total knee replacement

## Abstract

Background

Total knee arthroplasty has become very popular globally as a safe surgical modality for relieving pain and improving functional outcomes in patients who fail to respond to conservative treatments; however, it may be associated with postoperative complications.

The aim of this study is to determine the incidence of postoperative complications occurring within the first 30 days after total knee replacement (TKR).

Materials and methods

This is a prospective cross-sectional study. All consecutive patients who underwent primary unilateral or bilateral total knee arthroplasty between November 2020 and July 2021 were included in the study. Patients were followed for a period of 30 days, and postoperative complications (if any) were documented. Continuous variables were expressed as means ± standard deviations. Categorical variables were expressed as frequency and percentages, and chi-square test was used to compare the qualitative variables. Univariate and multiple logistic regression analyses were done to analyze the magnitude of associations of the complication with other predictor variables keeping a level of significance of <0.05.

Results

The overall complication rate within the 30-day window was 7.0%. Postoperative surgical site infections (SSI) were noted in three patients (2.6%). Thromboembolic complications were seen in only one patient (0.9%). One patient (0.9%) was readmitted within the one-month period after initial discharge, and one patient (0.9%) expired within 12 hours postoperatively.

Conclusion

TKR renders satisfactory results with a low incidence of complications in general; however, wound infections, thromboembolic complications, and cardiovascular complications do occur postoperatively. Male gender, obesity, and bilateral TKRs remain the notable risk factors for the development of complications post-procedure.

## Introduction

Total knee arthroplasty (TKA) has become a popular surgical option for patients who fail to respond to conservative treatments. TKA is a cost-effective procedure and is growing globally at a considerable rate [1]. Majority of patients who undergo TKA report pain relief and improved functional outcomes [2-4]. TKA is generally a safe procedure; however, it may be associated with postoperative complications that result in suboptimal clinical outcomes, increased financial burden, disability, and mortality [5,6]. The majority of postoperative complications following total joint replacements in the lower extremities occur during the hospital stay [5].

The risk of mortality is increased in the setting of cardiovascular disease, old age, simultaneous bilateral arthroplasty, and the use of cemented implants [7]. The risk of mortality associated with TKA is low, ranging from 0.1% to 0.8%, and is on a declining trajectory over the last decade [7,8]. This declining trend in postoperative mortality is likely due to improvements in patient selection and better perioperative care [8]. Perioperative morbidity after TKA is mainly related to infection and thromboembolism. Postoperative infection is an important cause of implant failure and revision arthroplasty [7,9]. The rate of postoperative infection has reduced from 9.1% in the 1980s to 1%-2% in the last decade [7,9,10]. The causative microorganisms are *Staphylococcus aureus*, *Staphylococcus epidermidis*, Group B *Streptococcus*, and *Pseudomonas aeruginosa* [7,11]. Periprosthetic infection is usually managed with one or more antibiotics, washout, debridement, revision, and arthrodesis. The rate of deep-seated infection is generally lower [12]. Patients with deep infections are treated with the removal of implants. TKA is associated with a low risk of thromboembolic ranging from 2% to 3% and may require prolonged use of anticoagulants.

The statistics on mortality and morbidity associated with TKA procedures are readily available from western nations, whereas locoregional data on this subject from countries like Pakistan is scarce. This study aims to determine the incidence rates of mortality and morbidity after TKA as well as identify risk factors and leading causes of complications in our region.

## Materials and methods

This prospective, cross-sectional, single-center study was conducted after approval from the Institutional Ethical Review Committee at the Section of Orthopedics, Department of Surgery, Aga Khan University Hospital, Karachi, Pakistan, and the approval number is 2022-5371-14526.

All consecutive patients enlisted for primary unilateral or bilateral TKA between November 2020 and July 2021 were included in the study. Indications of surgery were osteoarthritis and rheumatoid arthritis. Surgeries were performed by five different surgeons, all of whom had more than 10 years of experience in arthroplasty. Patients with revision total knee and those who lost to follow-up were excluded.

All patients underwent cemented TKA implants, and tourniquets were used in all cases. A standard midline medial parapatellar approach was used in all procedures. Low molecular weight heparin (enoxaparin) was administered for thromboprophylaxis 12 hours before surgery, and Ascard or enoxaparin was then continued until two weeks after surgery. Perioperative antibiotics (cefazolin or ciprofloxacin) were used prophylactically for 48-72 hours postoperatively.

All patients were followed in routine clinical visits for a period of two weeks and one month postoperatively and thereafter. Data items collected for each patient included demographics; pre-procedural comorbidities; length of hospital stay; any complications such as infection; thromboembolic events including deep venous thrombosis, pulmonary embolism, and cardiac event; and readmission within the 30-day interval following TKA.

Data analysis was done on Statistical Package for the Social Sciences (SPSS) version 23 (IBM Corp., Armonk, NY). Continuous variables were expressed as means + standard deviations. Categorical variables were expressed as frequency and percentages, and chi-square test was used to compare the qualitative variables. Univariate and multiple logistic regression analyses were done to analyze the magnitude of associations of the complication with other predictor variables keeping a level of significance of <0.05.

## Results

A total of 114 patients were included in the final dataset with a mean age of 62.8 ± 8.99 years (range: 24-82 years). Majority of the patients were females, accounting for 76.3%, while 23.7% were males. Eighty five out of the total 114 patients had prior comorbid conditions. Among them, 66.7%, 36.8%, and 25.7% had hypertension, obesity, and diabetes mellitus, respectively (Table 1).

**Table 1:** Patient demographics

Age (in years)	62.8 ± 8.99
Gender	
Male	23.7%
Female	76.3%
Comorbidities	
Hypertension	66.7%
Diabetes mellitus	25.7%
Obesity	36.8%

In total, 39 patients underwent unilateral TKA, and 75 patients underwent one-stage bilateral TKA. Majority of the patients constituting 74.6% had ASA (American Society of Anesthesiologists) level 2; 14% and 11.4% of patients had ASA levels 3 and 1, respectively. Out of the total 114 patients, 101 underwent TKA under general anesthesia and 13 under spinal anesthesia (Table 2).

**Table 2:** Surgery characteristics ASA: American Society of Anesthesiologists.

Laterality	
Unilateral	34.2%
Bilateral	65.7%
ASA status	
I	11.4%
II	74.5%
III	14%
Anesthesia	
General	88.59%
Spinal	11.4%

The overall complication rate within the 30-day window was 7.0%. Postoperative surgical site infections (SSI) were noted in three patients (2.6%). Among these, two patients had superficial infections, and one patient had deep-seated infections. Thromboembolic complications were seen in only one patient (0.9%) who was diagnosed with pulmonary embolism one week after the surgery. Cardiopulmonary complications were seen in two patients accounting for 1.8%. One patient (0.9%) was readmitted within the one-month period after initial discharge, and one patient (0.9%) expired within the 12 hours postoperative period due to sudden cardiac death (Table 3).

**Table 3:** Postoperative complications in a period of 30 days

Classification	Complication	Number	Percentage
Systemic complications	Thromboembolic complications	1	0.9%
	Cardiopulmonary complications	2	1.8%
Local/regional complications	Superficial surgical site infection	2	1.8%
	Deep wound infection/organ space infection	1	0.9%
	Readmission	1	0.9%
Others	Death	1	0.9%
Total		8	7%

The complication rate was 33.3% in males and 14.4% in females with a p-value of 0.043, which is statistically significant. The complication rate was 9.5% for unilateral TKA and 23.6% for bilateral TKA, and the differences were statistically significant (p-value = 0.080). There was no significant difference in the frequency of complications in the study population based on the type of anesthesia received during the surgery (p-value = 0.37). Patients with comorbidities had a slightly higher rate of complications of 19.4% versus 12.5% in the subgroup without comorbidities, which was not statistically significant (p-value = 0.732). No significant differences were observed in the patient subgroups based on their ASA scores (p-value = 0.75).

Univariate logistic regression analysis was performed for separate complications, i.e., deep vein thrombosis (DVT), pulmonary embolism (PE), SSI, and cardiac complications. Interestingly, the odds of the postoperative length of hospital stay of less than three days and more than seven days among patients with SSI were 21 times and 34 times more as compared to those who did not have SSI (p = 0.037). The odds of obesity among patients with SSI were 3.5 times more as compared to patients who did not have SSI (p = 0.048). A multiple logistic regression model was then created, which showed the odds of obesity are higher among patients with SSI as compared to those who did not have SSI keeping all other variables constant (OR = 3.6, CI: 1.9-6.5). In the same model, we identified that the odds of patients with longer length of stay (>7 days) or decreased length of stay (<3 days) are higher among the patients with SSI as compared to those who did not have SSI (OR = 31 and 23, CI: 13.8-48 and 8.3-40).

## Discussion

In our study, the incidence of 30-day postoperative complications rate is 7% including local and systemic complications. Previous literature suggests that although the complications after TKA seldom occur, they are dreaded [2,3]. According to a study by Belmont et al., 1.83% of the total subjects had to experience major systemic complications including events that were cardiovascular in origin, and 3.20% of patients had minor systemic complications including urinary tract infections and deep venous thrombosis, while 1.43% of the patients experienced local complications [2]. The most common postoperative complication encountered in our data set is SSI. This was seen in 2.7% of the patient population, with superficial site infection and deep site infection accounting for 1.8% and 0.9%, respectively. Belmont et al. reported that out of all the study subjects, 0.79% of the patients had superficial site infection and 0.30% of the patients experienced deep wound infection [2]. In a study by Seah et al., out of 2219 patients, SSI was reported to be 1.8%, out of which 1.44% of the subjects had superficial site infection, and 0.36% of the patients had deep wound infection [7]. Feng et al. reported the incidence of 30 days postoperative wound infections as 0.8%, with deep and superficial infection accounting for 0.1% and 0.7%, respectively [13].

The present study further discovered that male gender, BMI ≥ 30.0 kg/m^2^, and bilateral procedures are significant risk factors for the development of complications within 30 days following TKA, which is consistent with the previous literature [2,5,13]. According to the results of our study, the complication rate was higher in males than females accounting for 33.3% and 14.4%, respectively, with a p-value of 0.043. Singh et al. also reported similar results with the postoperative complications being significantly higher in males (6.18%) than females (5.26%) with a p-value of 0.010 [14].

Thromboembolic phenomena are a recognized complication in the postoperative period after lower limb arthroplasty mostly occurring in the period of 5-36 days, eventually resulting in increased morbidity and mortality. In our study, none of the patients were reported to develop DVT within the first 30 days post-TKA; however, one patient (0.9%) was diagnosed with PE on imaging studies after one week of TKA. This rate is quite similar to the previous literature. Feng et al. in their study reported that PE was seen in 0.3% of cases [13]. Similarly, in a study by Belmont et al., the incidence rate of PE was reported as 0.78% [2]. Aggressive VTE prophylaxis, both pharmacological and mechanical, remains the only solution to prevent the occurrence of DVT and PE [15]. A meta-analysis by Lee et al. reported that the incidence of PE in the Asian population was quite low accounting to be 0.01% [16].

There is a considerable risk of postoperative mortality following major surgeries; however, TKA remains an optimal procedure with a rare incidence of postoperative mortality [11,17]. Belmont et al. and Seah et al. reported the 30-day mortality rate after total knee replacement (TKR) as 0.1% and 0.27%, respectively [2,7]. Parvizi et al. reviewed 22,540 patients in the Mayo Clinic and reported a 30-day mortality of 0.24%, and the decreasing trends of mortality rates have been recorded in the past three decades [5]. In our study, there was only one mortality out of the total 114 subjects accounting for 0.9%, which occurred due to sudden cardiac arrest in the first 24 hours after the surgery. The findings of our study support the previous literature, which suggests that cardiovascular diseases are one of the major causes of mortality after TKA. Cardiac health concerns must be addressed, and the patients should be optimized before proceeding with surgery. In a study by Smith et al., the postoperative mortality rate was found to be 0.08% with myocardial infarction being the most common cause of death after TKA [18]. Chan et al. in their study reported that 50% of the deaths in their dataset occurred due to cardiovascular diseases [19]. The existing literature also emphasizes the fact that there is a declining trend of postoperative mortality over the past years. Harris et al. in their study found that the 30-day mortality was reduced from 0.17% in 2003 to 0.08% in 2017 [20].

A strong positive association was found between SSI and patients with higher BMI on the multiple logistic regression model (OR = 3.6, CI: 1.9-6.5); this finding is consistent with the pre-existing literature. In a study by Wallace et al., the results of multivariate analysis showed a positive association between wound infection and patients with a BMI of 30 kg/m^2^ and over (30-35 kg/m^2^ adjusted OR = 1.23; 95% CI: 1.01-1.50; P = 0.04; >35 kg/m^2^ adjusted OR = 1.39; 95% CI: 1.11-1.72; P < 0.01) [21].

According to the previous literature, TKA is regarded to be one of the safest and most cost-effective procedures for patients with osteoarthritis, and it yields clinically successful outcomes with patients returning to their routine activities. This study has a few limitations. The results of our study are not generalizable to the entire population as it is a single-center study, and a greater number of TKA procedures are performed in more affordable healthcare settings in Pakistan. At our institution, the number of yearly procedures performed is much lower due to a relatively higher financial cost burden. We aim to collaborate with other tertiary care hospitals and publish results from multi-institutional analysis in a future attempt.

## Conclusions

To conclude, TKR renders satisfactory results with a low incidence of complications in general; however, wound infections, thromboembolic complications, and cardiovascular complications do occur postoperatively. Male gender, obesity, and bilateral TKRs remain the notable risk factors for the development of complications post-procedure. The incidence of postoperative complications can be reduced to a considerable extent if the patients are optimized preoperatively and sterile measures are followed properly during the surgery.
